# Effect of Extra Virgin Olive Oil and Butter on Endothelial Function in Type 1 Diabetes

**DOI:** 10.3390/nu13072436

**Published:** 2021-07-16

**Authors:** Antonio Cutruzzolà, Martina Parise, Rosarina Vallelunga, Francesco Lamanna, Agostino Gnasso, Concetta Irace

**Affiliations:** 1 Dipartimento di Medicina Sperimentale e Clinica, University Magna Græcia, 88100 Catanzaro, Italy; a.cutruzzola@unicz.it (A.C.); vallelungarosarina@hotmail.it (R.V.); sisco.lamanna@gmail.com (F.L.); gnasso@unicz.it (A.G.); 2 Dipartimento di Scienze della Salute, University Magna Græcia, 88100 Catanzaro, Italy; parise.martina@gmail.com

**Keywords:** EVOO, butter, dietary fat, Mediterranean diet, endothelial function, flow-mediated dilation, gastric emptying, type 1 diabetes, high glycemic index diet

## Abstract

Post-prandial hyperglycemia can be relevant in developing early manifestations of atherosclerosis. EVOO (Extra Virgin Olive Oil), rich in saturated fatty acids and commonly used in the Mediterranean diet, seems to control post-prandial hyperglycemia better than butter. Subjects with type 1 diabetes are at higher risk of developing cardiovascular disease and show endothelial dysfunction, an early manifestation of atherosclerosis in the first years of the disease. Our study aims to evaluate whether EVOO and butter influence endothelial function in subjects with type 1 diabetes when added to a single high glycemic index (HGI) meal. In this exploratory cross-over study, 10 subjects with type 1 diabetes and 6 healthy subjects were scheduled to receive two types of HGI meals: one enriched with EVOO and one with butter. Before and after each test meal at different time points, all subjects underwent the evaluation of endothelial function by flow-mediated dilation technique, glucose and lipids measurements, and gastric emptying assessment by ultrasound. Flow-mediated dilation significantly increased after EVOO-enriched meal compared with butter in subjects with type 1 diabetes (two-way-repeated measurements ANOVA, *p* = 0.007). In patients with type 1 diabetes, the add-on of EVOO to HGI meal improves vascular function compared to butter, which has detrimental effects.

## 1. Introduction

Post-prandial hyperglycemia can be relevant in developing early manifestations of atherosclerosis. The acute post-prandial glucose fluctuation increases oxidative stress and impairs vascular function by mainly reducing nitric oxide (NO) bioavailability, one of the most potent antiatherosclerotic and vasoactive substances [1,2].

Despite improved insulin treatment and technology, post-prandial glycemic control is still a challenge in type 1 diabetes (T1D). 

Many factors such as meal composition, quantity and quality of macronutrients, pre-meal insulin administration, and gastric emptying rate (GER) influence post-prandial glucose excursion [3,4]. Though carbohydrates are the primary macronutrient affecting post-prandial hyperglycemia, fat, protein, and glycemic index may further contribute [5].

The chronic consumption of saturated fatty acids (SFA) impairs endothelial function and contributes to the so-called post-prandial dysmetabolism [6,7,8]. In contrast, the monounsaturated (MUFA) and polyunsaturated (PUFA) fatty acids improve post-prandial metabolic control in healthy subjects and patients with type 2 diabetes [9]. One of the predominant sources of MUFA is EVOO (Extra Virgin Olive Oil), a common ingredient of the Mediterranean diet. Regular EVOO consumption reduces the incidence of macrovascular complications, prevents the onset of diabetes in high risk subjects, and downregulates the synthesis of C reactive protein, interleukin-6, and other pro-inflammatory molecules probably through the effect of polyphenols, known to have antioxidant capacity as the downregulation of pro-inflammatory gene expression and the inhibition of synthesis and release of inflammatory proteins [9,10,11,12]. A single EVOO-enriched high glycemic index meal (HGI) blunts post-prandial hyperglycemia, while the effect on endothelial function in T1D has not been explored [13]. No data are available about the vascular impact of EVOO in T1D.

This led us to evaluate the effect of EVOO consumption on endothelial function. The endothelial function in vivo, with non-invasive methods, can be evaluated through the variations in the diameter of an arterial vessel following an increase in blood flow velocity in the same vessel. Currently, the most frequently used method involves measuring the diameter of the brachial artery after ischemia of the forearm induced by a pneumatic cuff [14,15]. This technique causes a sudden and large increase in flow velocity, which is responsible for subsequent changes in diameter. Alternatively, the increase in blood flow velocity can be induced by moderate handgrip exercise. The two tests not only use different stimuli but probably also activate different metabolic pathways. Therefore, their combined use can provide more complete information on the function of the endothelium [16].

We have designed our study to test the hypothesis that EVOO might have a beneficial effect on endothelial function compared to butter in subjects with T1D in the contest of an unfavorable meal, like the HGI meal [17].

We have also measured post-prandial glucose and lipids, which may affect endothelial function and GER, to evaluate if EVOO or butter may influence the gastric emptying rate.

## 2. Materials and Methods

Subjects and study protocol: The present is an exploratory study with a cross-over design approved by the local Ethical Committee, Comitato Etico Regione Calabria Area Centro, Nr 302/2018. Patients with T1D were recruited at the diabetes care center. Inclusion criteria were signed informed consent, age ≥ 18 years, diagnosis of T1D since at least one year, HbA1c < 8.5% (69 mmol/mol), stable insulin therapy for at least three months before the enrolment into the study. Exclusion criteria were: pregnancy, celiac disease, history of cardiovascular diseases, hypertension, hyperlipidemia, retinopathy, nephropathy, neuropathy, and ongoing treatment with vasoactive drugs. Clinical information, regularly collected according to national guidelines, were retrieved from electronic medical records [18]. Control subjects were recruited among students attending the Medical School. For controls, who were not diabetic, the exclusion criteria were the same as those for patients.

All patients and control subjects, who met inclusion and exclusion criteria, and signed the informed consent, were scheduled for the two study visits one week apart. Each participant received two types of HGI meal: one enriched with EVOO and one with butter. The sequence of the two meals was randomly assigned. All participants were asked to have a light dinner the day before the study and an early light breakfast (6.30 am) on the same day to avoid the second-meal effect [19]. They were also asked to refrain from cigarettes and alcohol from midnight and avoid strenuous physical activity before and in the morning of the test meal. Participants consumed the test meal at lunchtime (12.30 pm) in 15 min, under investigators’ supervision, and then remained fasting until the end of the study procedure. Capillary blood glucose was measured in patients with type 1 diabetes before each test meal by Accuchek Aviva meter. The study was rescheduled for a different day in case of blood glucose outside the range 70–180 mg/dL (3.9–10.0 mmol/L). The rapid-acting insulin was given immediately before each meal as suggested by the summary of product characteristics and according to the amount of carbohydrates. T1D patients in multiple daily insulin injections (MDI) were invited to inject long-acting insulin the evening before the study. Those in continuous subcutaneous insulin infusion (CSII) continued basal insulin infusion. Before the first test meal, all participants underwent height, body weight, and waist circumference measurements. Body Mass Index (BMI) was calculated according to the formula weight/height^2^ (kg/m^2^). Blood pressure (systolic blood pressure SBP; diastolic blood pressure DBP) and heart rate (HR) were also measured twice after a rest of at least 5 min and the average calculated. Blood samples were collected before each test meal (pre-meal) and 1 h, 2 h, 3 h, 4 h, and 5 h post-meal.

The vascular study evaluating endothelial function was performed before each test meal (pre-meal) and 1 h, 3 h, and 5 h post-meal. The 1 h was selected to evaluate the occurrence of the FMD change at the post-prandial hyperglycemic peak [20,21]. The 3 h and 5 h were set in an arbitrary way to assess the rate at which post-prandial FMD returns to pre-prandial values if changed.

The gastric emptying rate (GER) was measured before and at 15 min, 90 min, 120 min, 180 min, 240 min, 300 min after the end of the meal.

Test meal: The HGI test meal was prepared by an expert dietitian and consumed in the vascular laboratory without heating, likely affecting the properties of the food. EVOO- and butter-enriched meals were the same in terms of energetic content (900 kcal) and macronutrient composition: carbohydrates 43%, protein 17%, and fat 40%. Fat differed in quality according to the amount of PUFA, MUFA, and SFA (Table 1). 

In detail, the test meal consisted of 80 g of white rice, 200 g of potatoes, 140 g of lean beef, a glass of water (100 mL), and 35 g of EVOO or 40 g of butter. EVOO and butter were added uncooked at the end of the meal preparation.

Blood analyses: Blood samples were collected before and during the test meal using a peripheral venous line. Blood glucose, triglyceride, total, and HDL cholesterol were measured with commercially available kits. HbA1c was measured by high-performance liquid chromatography (HPLC) aligned with DCCT only in T1D patients on the day they signed the consent.

Endothelial function: Endothelial function was evaluated by the FMD technique to measure the dilation of the brachial artery after ischemia (reactive hyperemia) induced by cuff inflation and after handgrip exercise [16,22]. The vascular studies were performed in a quiet and temperature-controlled room after 10 min of supine rest. Premenopausal women were assessed in the first week of the menstrual cycle to minimize the effect of sex hormones on vasodilation [23]. The sonographer was blinded to the type of test meal. The brachial artery was imaged ~3–4 cm above the elbow in the longitudinal section on the anterior side of the biceps muscle keeping the angle between the ultrasound beam and the vessel at 90°. After optimizing the image, by fine-tuning the gain, the brachial artery internal diameter (ID) was measured before cuff inflation and exercise (baseline ID) as suggested by the guidelines [16,22]. Brachial artery ID was defined as the distance between the intima–lumen interface of the near wall and the lumen–intima interface of the far wall. The ischemic test was carried out as the first vascular test by inflating a pneumatic cuff around the forearm up to 250 mmHg and maintaining inflation for 5 min. Images of the brachial artery ID were measured at baseline, 1, 2, and 3 min after the cuff release [24,25]. The three set times were previously determined in a mean age sample of 28 years [26]. The peak FMD, defined as the highest value among the three measurements, was used for the statistical analyses.

Handgrip exercise was performed as the second vascular test, as soon as the brachial artery diameter returned to baseline, by using the dynamometer T.K.K. 5401 (Takei Scientific Instruments Co., LTD., Niigata-City, Japan). First, subjects were invited to complete a maximum voluntary contraction (MVC) three times. The mean of the three attempts was used to establish the MVC of each participant. Participants were then invited to perform 5 min of rhythmic isometric handgrip (2 s contraction: 3 s relaxation ratio) at 30% intensity of MVC. Brachial artery ID was measured at the 5th minute of exercise.

The 30% MVC represents the maximal contraction, ensuring adequate vasodilation without heart rate and blood pressure change and the appearance of pain or fatigue. Furthermore, 5 min of 30% MVC handgrip exercise represents the time to reach the maximal vasodilation [26,27].

Ischemia and handgrip exercise explore the endothelium-dependent dilation following the increased metabolic demand. The common mechanism inducing brachial artery vasodilation is the decrease in peripheral vascular resistances occurring during ischemia and exercise and the increase in arterial blood flow velocity occurring after cuff deflation and during exercise. We have decided to use two vascular tests for two reasons. One is the different intensity and duration of the stimulus, vigorous and transient after ischemia and less intensive and sustained after exercise. The other is the different vasodilatory response to the two stimuli in the same individual [28], possibly due to the activation of different pathways.

The vascular tests were performed with a Philips HD 11XE Ultrasound machine (Royal Philips Electronics, Bothell, WA, USA) equipped with a 12-3 MHz linear array probe. 

All images of the brachial artery were recorded during the study and evaluated offline by dedicated software (AUTODESK Design Review, BSA, Italy, https://knowledge.autodesk.com/it (accessed on 16 May 2018)). ID was measured in 1 cm lengths at three different locations of the artery. The mean value of the three measurements was used for the analyses.

FMD after ischemia and exercise has been expressed as the percentage of dilation from baseline to each observation time and calculated as follows: (((brachial artery ID at 1 min after ischemia or 5th minute during handgrip exercise—baseline ID)/baseline ID) × 100). 

Gastric emptying rate: GER was evaluated by the ultrasound-based technique, a valid and non-invasive alternative to scintigraphy [29]. The emptying rate was assessed at the gastric antrum level that provides the most reliable quantitative information for gastric volume compared to the fundus area. The cross-sectional antral area correlates with volume up to 300 mL in a close-to-linear fashion [30]. The ultrasound study was performed with a Philips HD 11XE Ultrasound machine (Royal Philips Electronics, Bothell, WA, USA) and a 5.0 MHz curved array probe. Participants were examined in the seated position with an angle of 45 degrees. The sonographer was blinded to the type of test meal. The probe was positioned to obtain a parasagittal image of the gastric antrum with the superior mesenteric vein, the abdominal aorta, and the left lobe of the liver used as reference points. The two-dimensional antral area (cm^2^) was automatically measured by the machine after outlining the circumference of the antrum with the caliper. At any observation time, GER was expressed as a percentage of emptying using the following formula = 100 − ((At/Amax) × 100) where At is the area measured at a given time point, and Amax is the maximum antral area recorded after meal ingestion. 

Outcomes: The primary endpoint of the study was to evaluate FMD after two different HGI meals enriched with EVOO and butter. As secondary endpoints, we assessed if the two types of fat would have a different impact on post-prandial glucose, lipids, and GER.

Statistical Analysis: Statistical analysis was performed using SPSS software ver. 25.0 (IBM, Armonk, NY, USA). Normal distribution was assessed graphically and with the Shapiro-Wilk test. Only triglycerides were not normally distributed, and a logarithmic transformation was approached. The two-tailed *t*-test for independent samples, the chi-square test, and the Fisher exact test were applied to compare continuous and categorical variables between the two groups (subjects with diabetes and controls), as appropriate. The FMD difference between patients with type 1 diabetes and control subjects after adjustment for sex, age, and smoking habit was evaluated by the ANCOVA (ANalysis of COVAriance). A two-way (within-within: time per meal interaction) repeated measures ANOVA (ANalysis of VAriance) was used to compare FMD, GER, glucose, and lipids after EVOO and butter in patients with type 1 diabetes and control subjects. To evaluate the overall FMD response to each test meal, we calculated the net incremental AUC (iAUCnet), applying the trapezoidal rule for all positive and negative increments. The AUC of glucose in response to meals and the AUC of GER was calculated as incremental AUC (iAUC) by including all the areas over the baseline and ignoring areas beneath the curve [31]. The relationship between the iAUC of post-prandial glycemia and the iAUCnet of FMD was assessed by a way of repeated measures of regression analysis. The relationship between the GER and iAUC of post-prandial glycemia was evaluated by the simple regression analysis, using the absolute difference of iAUC of blood glucose measured after butter and EVOO and the absolute difference of iAUC of GER after butter and after EVOO.

For all analyses, a two-sided *p*-value < 0.05 was considered statistically significant.

Sample size: Sample size was computed for a within-within (meal per time interaction) repeated measures design, with FMD as the outcome. Based on preliminary data, in the hypothesis of a 4% difference in vasodilation between the two types of test meals, the number of subjects to be enrolled was 9 in each group to achieve a power = 80% and a level of significance = 0.05.

## 3. Results

Ten patients with T1D and six healthy control subjects were enrolled in the study. Table 2 shows the clinical characteristics and metabolic parameters of the participants. 

T1D patients had significantly higher SBP, pre-meal glucose, and significantly lower triglycerides compared to controls. Age, male sex, waist, BMI, DBP, HR, smoke habit, total cholesterol, HDL- and LDL-cholesterol were not significantly different. The prevalence of male sex and smokers was higher in control subjects.

We have first compared sex- and smoke habit-adjusted baseline FMD of T1D patients and control subjects. The results were the following: FMD after ischemia, T1D 8.0 ± 0.6%, controls 10.5 ± 0.8% (mean ± SE), *p* < 0.0001; FMD after handgrip exercise, T1D 7.2 ± 0.8% vs. controls 11.0 ± 1.0% (mean ± SE) *p* = 0.0001. All participants had the peak FMD at 1 min from the cuff deflation.

**Figure 1:**
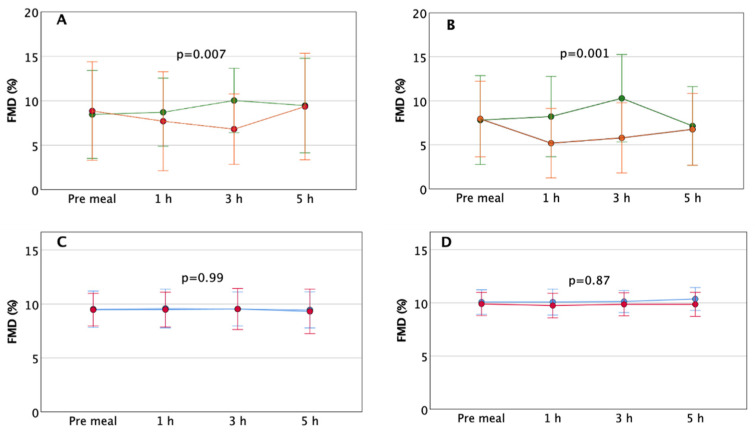
Flow-mediated dilation (FMD) before and after EVOO- and butter-enriched test meals in T1D and control subjects. (**A**) FMD after ischemia in T1D (green EVOO, orange butter); (**B**) FMD after handgrip exercise in T1D (green EVOO, orange butter); (**C**) FMD after ischemia in control subjects (blue EVOO, red butter); (**D**) FMD after handgrip exercise in control subjects (blue EVOO, red butter).

In Figure 1 we have displayed FMD at each time point for each meal in T1D and control subjects. The two-way repeated-measures ANOVA revealed a significant FMD increase over time after EVOO compared to butter in T1D. No difference was detected in control subjects. 

**Figure 2:**
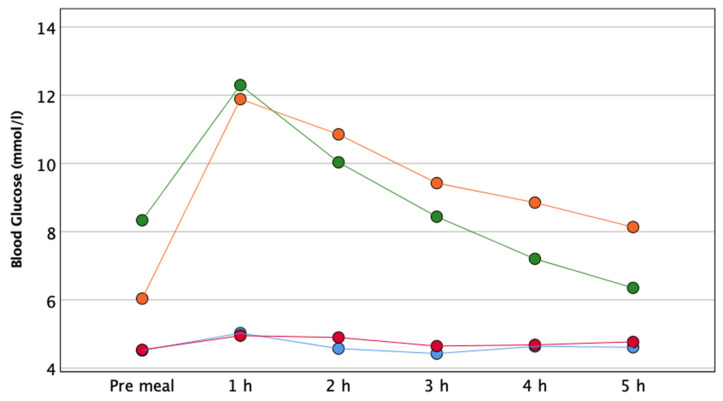
Blood glucose at pre-meal, 1 h, 2 h, 3 h, 4 h, 5 h after the two test meals in T1D and healthy controls. Green and orange represent T1D after EVOO- and butter-enriched meals; blue and red represent controls after EVOO- and butter-enriched meals.

The mean change in blood glucose over time is presented in Figure 2. Blood glucose was significantly lower after EVOO than butter in T1D, while it was almost unchanged in control subjects (two-way repeated-measures ANOVA: T1D *p* = 0.003; controls *p* = 0.56).

The glucose iAUC 0–5 h was also significantly lower after EVOO compared to butter in T1D (520 ± 474 vs. 1174 ± 730 mmol/L, *p* < 0.005), and comparable in control subjects (EVOO 88 ± 46 vs. butter 91 ± 89 mmol/L, *p* < 0.94).

Pre-meal glucose was slightly higher before EVOO than butter in T1D, though the difference was not statistically significant (EVOO 8.3 ± 2.7 mmol/L vs. butter 6.0 ± 2.0 mmol/L, *p* = 0.06).

The mean calculated insulin dose, injected before two meals, was 8.4 ± 3.9.

No significant relationship between blood glucose and FMD was found in T1D. In detail, EVOO: iAUCnet FMD after ischemia and iAUC blood glucose: r^2^ = 0.09, *p* = 0.40; iAUCnet FMD after exercise and iAUC blood glucose: r^2^ = 0.13, *p* = 0.17; butter: iAUCnet FMD after ischemia and iAUC blood glucose: r^2^ = 0.27, *p* = 0.12; iAUCnet FMD after exercise and iAUC blood glucose: r^2^ = 0.06, *p* = 0.50.

**Figure 3:**
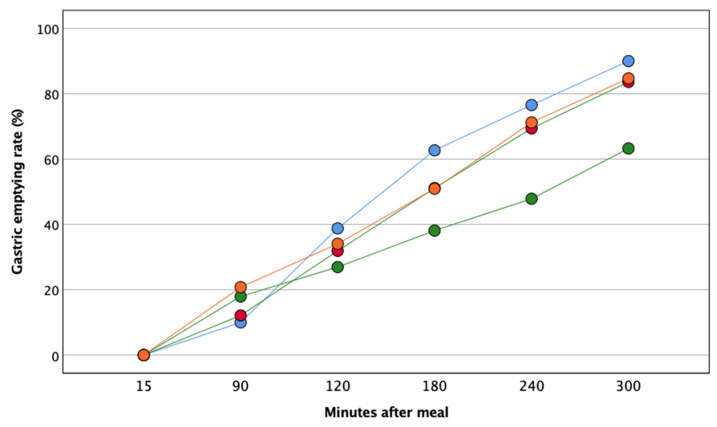
Gastric emptying rate evaluated by ultrasound in T1D and healthy controls after the two meals. Green and orange circles represent T1D after EVOO- and butter-enriched meals; blue and red circles represent controls after EVOO- and butter-enriched meals.

GER measured at pre-meal and 15 min, 90 min, 120 min, 180 min, 240 min, 300 min post-meal is displayed in Figure 3. 

GER was significantly slower after EVOO than butter in T1D, while, as well as for blood glucose, no statistically significant difference was found in control subjects.

To evaluate a possible relationship between GER and post-prandial glucose excursion in subjects with T1D, we correlated the absolute difference between EVOO and butter of iAUC blood glucose and iAUC GER across five hours. The r^2^ was 0.42 and the *p*-value 0.06. 

Lipids did not differ in T1D and control subjects after EVOO and butter (Table 3).

## 4. Discussion

The present study provides the first evidence that EVOO enhances endothelial function when added to a single meal with HGI compared to butter in patients with T1D. The EVOO-enriched test meal increases FMD by 18% after ischemia and 32% after exercise than baseline FMD, while butter decreases FMD by 22% after ischemia and 34% after exercise.

In control subjects, the endothelial function is not influenced by EVOO or butter.

Before discussing the results of this work, we believe it is helpful to comment on two methodological aspects. First, we chose a diet with a high glycemic index for this study because we believe it is most likely to cause alterations in endothelial function. Second, we have selected two different methods for the evaluation of endothelial function, one based on a maximal stimulus, such as that induced by ischemia, and the other based on a much more physiological stimulus, such as that of physical exercise, in an attempt to intercept any alteration in the function of the endothelium.

Although the baseline FMD value in patients with type 1 diabetes cannot be considered abnormal (8% and 7% after ischemia and exercise), it is significantly lower than control subjects. Clinical studies have demonstrated a significant 8–13% higher cardiovascular risk for each percent point decrease in brachial artery FMD [32,33].

The enhancement of endothelial function by EVOO observed in the present study is not attributable to the blunted post-prandial hyperglycemia. Indeed, no relationship between blood glucose and FMD, measured after ischemia and exercise, arises. The observed benefit and harm might be attributable to the content of MUFA, PUFA, and polyphenols in EVOO and SFA in butter. In vitro studies have demonstrated that oleic acid reduces the expression of inflammatory adhesion molecules by the endothelial cells, and polyphenols stimulate the synthesis and release of NO and protect the endothelium from the post-prandial glucose-induced oxidative stress [9,10]. Conversely, the detrimental effect on endothelial function after the butter-enriched test meal could be caused by the downregulation of NO-mediated by the SFA [34,35]. In vivo studies have demonstrated that the assumption of an undesirable amount of SFA associate with a higher rate of cardiovascular diseases in subjects at high risk to develop diabetes and endothelial dysfunction in overweight subjects with type 2 diabetes [36,37].

The comparison of studies evaluating the impact of different macronutrients on endothelial function is quite tricky because the potential beneficial or detrimental effect depends on the main characteristic of the meal and the combination of nutrients. We have decided to evaluate the addition of two different fat in the context of an HGI meal that is not advisable in patients with T1D but common in several countries. It might be argued that the amount of EVOO added to the test meal we administered to patients with T1D is high and likely unfavorable in the long term. However, the amount we used is comparable to that used in similar researches as the PREDIMED study, which has demonstrated that the chronic use of 50 mL of EVOO, or the equivalent of nuts, reduces by 30% the risk of cardiovascular events as myocardial infarction and stroke, and decreases the new onset of diabetes in high-risk subjects [12].

As additional findings, we find that EVOO blunts post-prandial hyperglycemia and slows down gastric emptying compared to butter. The two responses might be correlated. Indeed, the low rate of gastric emptying should delay the absorption of carbohydrates throughout the gut by increasing the GLP-1 release or decreasing the activity of the DPP-4 inhibitor [37]. Even though in our study the correlation between post-prandial hyperglycemia and GER is not statistically significant (*p*-value equal to 0.06), about 40% of the variation of post-prandial glucose excursion is attributable to gastric emptying. However, EVOO may account for the improved post-prandial hyperglycemia independently from the GER due to its intrinsic antioxidant properties improving the insulin signaling cascade [38,39].

As reported in the result section, we found a slight but not significant difference in pre-meal blood glucose between EVOO and butter test meals. However, a very recent article has demonstrated that pre-meal fasting blood glucose does not affect gastric emptying in subjects with T1D [40].

There is a complete absence of any effect of EVOO or butter on endothelial function in control subjects. Indeed, after ischemia and after exercise, flow-mediated vasodilation does not change at all over the 5 h, along with minimal changes in glucose and blood lipid concentration. We can speculate that the homeostatic mechanisms that guarantee a constant blood flow adequate to the metabolic needs are perfectly functioning in subjects without diabetes, at least in the forearm area. Similarly, the gastric emptying rate and the post-prandial glucose excursion do not seem to be affected by the addition of EVOO or butter.

We do not find, in both groups, any significant difference in post-prandial lipids when comparing the two test meals. As known, cholesterol is not influenced by a single meal, while triglycerides can vary based on the number of chylomicrons produced in the gut and the rate of catabolism in the systemic circulation by the activity of lipoprotein lipase. However, subjects enrolled in the study have a very low amount of triglycerides at baseline, either before EVOO and butter, suggesting a normal lipid post-prandial clearance.

The present study has some strengths and some limitations. The meals were prepared in the hospital. There was no interference by participants who were strictly monitored, the study protocol was completed in the same room under medical surveillance, any physical activity did not influence post-meal blood glucose, and the same dose of insulin was given to each subject before each test meal. The vascular study was performed by the same operator and keeping the same baseline conditions before taking the EVOO- and butter-enriched meal. The major limit is the number of control subjects recruited in the study that is lower than that calculated. Unfortunately, the lockdown due to the COVID-19 pandemic has restricted the access of volunteers to the research center. However, we believe that, according to the study design, the results obtained in subjects with T1D are consistent and reliable. As a potential limit, we must further consider the investigation of only the HGI test meal. This choice was driven by the evidence that safer meals as low carbohydrates and high fiber meal have a beneficial effect on post-prandial hyperglycemia and endothelial function [37]. HGI food as rice and potatoes, which are common among young people and in several countries, is responsible for high post-prandial hyperglycemia that is known to be an independent risk factor for cardiovascular events and lack of adequate glycemic control [41]. With respect to the technical procedure, and in the absence of any comparable research study, the selection of the observation times to evaluate FMD in the post-prandial state was established taking into account the time to perform both vascular procedures and to assess the rate at which FMD returned to the pre-prandial state. The internal diameter measurement was performed at three different time points and not continuously after the cuff deflation. Finally, we have not included the measurement of markers of NO bioavailability in the post-prandial state in the study design.

Our research has some clinical implications. EVOO, added to a single unfavorable meal, preserves endothelial function and blunts post-prandial hyperglycemia protecting the vascular tree. These results could suggest dietary modifications suitable for maintaining endothelial function.

## 5. Conclusions

The present findings demonstrate that patients with T1D have a significantly lower endothelium-mediated vasodilation compared to non-diabetic controls. The addition of EVOO enhances the vasodilatory capacity of the brachial artery even in the short term. The mechanisms by which EVOO and butter exert their effects are probably mediated by the intrinsic properties of the fats influencing the paracrine activity of the endothelial cells.

## Figures and Tables

**Table 1:** Energy content and macronutrient composition of the test meals.

Test Meal Composition	EVOO	Butter
Energy (kcal)	900	900
Carbohydrates (g)	100.1	100.5
Starches (g)	90.1	90.1
Proteins (g)	38.5	40.9
Fiber (g)	4.0	4.0
Fat (g)	41.1	39.7
Cholesterol (mg)	99.4	206.5
MUFA (g)	28.0	11.6
PUFA (g)	4.6	2.6
SFA (g)	7.7	21.7
Glycemic Index (%)	76	76

MUFA: monounsaturated fatty acids; PUFA: polyunsaturated fatty acids; SFA: saturated fatty acids.

**Table 2:** Anthropometric, clinical, and biochemical characteristics of subjects with type 1 diabetes and control subjects.

	Type 1 Diabetes (N 10)	Controls (N 6)	*p*
Age (years)	28 ± 8	25 ± 3	0.38
Male n (%)	7 (70)	6 (100)	0.25
Waist (cm)	82 ± 10	81 ± 5	0.91
BMI (kg/m^2^)	24.2 ± 3.0	22.3 ± 1.8	0.18
Systolic BP (mmHg)	122 ± 10	110 ± 10	0.04
Diastolic BP (mmHg)	73 ± 8	71 ± 10	0.68
HR (beats/min)	72 ± 10	70 ± 8	0.69
Smokers n (%)	1 (10)	3 (50)	0.12
Pre-meal glucose, (mmol/L) *	7.3 ± 2.9	4.5 ± 0.3	0.04
Pre-meal triglycerides (mmol/L) *	0.71 ± 0.24	0.98 ± 0.21	0.04
Pre-meal total cholesterol (mmol/L) *	4.07 ± 0.67	3.76 ± 0.65	0.38
Pre-meal HDL-cholesterol (mmol/L) *	1.17 ± 0.28	1.19 ± 0.31	0.90
Pre-meal LDL-cholesterol (mmol/L) *	2.43 ± 0.41	2.20 ± 0.36	0.92
Disease duration (years)	12 ± 6	-	-
HbA1c (%)	7.4 ± 0.8	-	-
Insulin daily dose (unit/kg)	0.56 ± 0.21	-	-

* Means of the values collected before the two vascular studies (ischemia and exercise). Data are expressed as mean ± standard deviation and categorical data as number/percentage.

**Table 3:** Lipids at pre-meal and after 1 h, 2 h, 3 h, 4 h, 5 h, post-meal in subjects with type 1 diabetes and control subjects.

Type 1 Diabetes	Pre Meal	1 h	2 h	3 h	4 h	5 h	*p*
EVOO total cholesterol (mmol/L)	4.05 ± 0.69	3.80 ± 0.56	3.89 ± 0.62	3.97 ± 0.72	3.89 ± 0.68	3.92 ± 0.65	*p* = 0.66
Butter total cholesterol (mmol/L)	4.00 ± 0.54	3.81 ± 0.55	3.82 ± 0.61	3.87 ± 0.53	3.96 ± 0.57	3.95 ± 0.57	
EVOO HDL-cholesterol (mmol/L)	1.15 ± 0.29	1.14 ± 0.27	1.12 ± 0.27	1.15 ± 0.29	1.13 ± 0.28	1.16 ± 0.28	*p* = 0.81
Butter HDL-cholesterol (mmol/L)	1.22 ± 0.25	1.17 ± 0.25	1.15 ± 0.26	1.17 ± 0.25	1.19 ± 0.25	1.18 ± 0.27	
EVOO LDL-cholesterol (mmol/L)	2.57 ± 0.58	2.27 ± 0.39	2.38 ± 0.49	2.39 ± 0.53	2.30 ± 0.44	2.29 ± 0.41	*p* = 0.23
Butter LDL-cholesterol (mmol/L)	2.43 ± 0.34	2.25 ± 0.34	2.24 ± 0.39	2.25 ± 0.31	2.28 ± 0.36	2.28 ± 0.37	
EVOO triglycerides (mmol/L)	0.72 ± 0.24	0.87 ± 0.26	0.87 ± 0.27	0.95 ± 0.31	1.02 ± 0.39	1.04 ± 0.49	*p* = 0.92
Butter triglycerides (mmol/L)	0.78 ± 0.32	0.86 ± 0.28	0.93 ± 0.35	0.99 ± 0.42	1.09 ± 0.47	1.06 ± 0.42	
**Control Subjects**	**Pre Meal**	**1 h**	**2 h**	**3 h**	**4 h**	**5 h**	
EVOO total cholesterol (mmol/L)	3.75 ± 0.65	3.78 ± 0.51	3.71 ± 0.66	3.76 ± 0.60	3.71 ± 0.60	3.79 ± 0.61	*p* = 0.85
Butter total cholesterol (mmol/L)	3.72 ± 0.45	3.64 ± 0.44	3.64 ± 0.41	3.65 ± 0.40	3.66 ± 0.47	3.77 ± 0.47	
EVOO HDL-cholesterol (mmol/L)	1.18 ± 0.30	1.16 ± 0.27	1.16 ± 0.29	1.18 ± 0.29	1.16 ± 0.30	1.19 ± 0.32	*p* = 0.86
Butter HDL-cholesterol (mmol/L)	1.24 ± 0.29	1.18 ± 0.32	1.19 ± 0.30	1.19 ± 0.30	1.20 ± 0.32	1.24 ± 0.37	
EVOO LDL-cholesterol (mmol/L)	2.11 ± 0.39	2.11 ± 0.19	2.08 ± 0.42	2.08 ± 0.41	2.07 ± 0.41	2.11 ± 0.36	*p* = 0.90
Butter LDL-cholesterol (mmol/L)	2.04 ± 0.44	1.94 ± 0.42	1.95 ± 0.39	1.87 ± 0.36	1.94 ± 0.41	1.99 ± 0.31	
EVOO triglycerides (mmol/L)	0.99 ± 0.22	1.12 ± 0.38	1.05 ± 0.09	1.10 ± 0.16	1.04 ± 0.19	1.07 ± 0.28	*p* = 0.69
Butter triglycerides (mmol/L)	0.98 ± 0.28	1.13 ± 0.29	1.09 ± 0.36	1.26 ± 0.99	1.12 ± 0.38	1.17 ± 0.29	

Data are expressed as mean ± standard deviation.

## Data Availability

The data presented in this study are available on request from the corresponding author. Most of the data are sensitive and not publicly available.
